# Editorial: The application of artificial intelligence in brain-computer interface and neural system rehabilitation

**DOI:** 10.3389/fnins.2023.1290961

**Published:** 2023-10-27

**Authors:** Fangzhou Xu, Dong Ming, Tzyy-Ping Jung, Peng Xu, Minpeng Xu

**Affiliations:** ^1^Qilu University of Technology, Jinan, China; ^2^Tianjin University, Tianjin, China; ^3^University of California, San Diego, San Diego, CA, United States; ^4^University of Electronic Science and Technology of China, Chengdu, China

**Keywords:** brain-computer interface, pattern recognition, artificial intelligence, brain function, neural computing

With the rapid growth of the global population, diseases related to motor dysfunction, such as stroke and spinal cord injury (SCI), are becoming an increasing challenge to public health. These diseases may lead to a series of functional declines such as cognitive impairment and emotional instability in patients, which seriously affects people's quality of life, and even endangers people's life and health, imposing heavy burdens on patients, families, and society. However, the pathogenesis of motor dysfunction-related diseases is complex, we currently lack effective and objective clinical diagnosis and intervention strategies. Artificial intelligence (AI) technology is developing rapidly, furthermore, it is attracting more attention from researchers and medical staff around the world in the brain-computer interface (BCI) and the clinical rehabilitation of motor dysfunction.

This Research Topic provides 19 papers on the application of AI techniques in the diagnosis and intervention of neurological diseases. The aim is to discover new algorithms, models, systems and applications that will facilitate the intersection of AI and neuroscience as well as promote the use of artificial intelligence in clinical medicine.

Innovations in classification recognition models and feature extraction methods improve performance on BCI systems. Li et al. proposes partial maximum correntropy regression (PMCR), a robust implementation of partial least squares regression (PLSR) using the maximum correntropy criterion. PMCR achieves better prediction and decoding performance compared to existing methods in noisy, inter-correlated, and high-dimensional decoding tasks. It minimizes neurophysiological pattern deterioration and improves electrocorticography decoding robustness for BCIs. Gao D-R. et al. introduces a novel unsupervised domain adaptation (UDA) approach. Effective data augmentation techniques are also explored. Experimental results demonstrate the superiority of the proposed method over state-of-the-art UDA methods in accuracy and MF1-Score. Gao D. et al. proposes a log-Mel spectrogram and convolution recurrent neural network (CRNN) model for fatigue detection using electroencephalogram (EEG) signals. Experimental results demonstrate accurate and stable detection performance, outperforming existing methods. This approach has potential for enhancing driver safety and accident prevention. Du et al. presents a single-trial P300 classification algorithm based on multi-person data fusion convolutional neural networks (CNN) to improve the efficiency and accuracy of P300 EEG signal classification. The algorithm outperforms single-person CNN classification and achieves higher accuracy with smaller models and fewer parameters. Zhang et al. focuses on the recognition of motor imagery (MI) EEG signals for the right upper limb and proposes a multi-branch fusion convolutional neural network (MF-CNN) that combines raw EEG signals and two-dimensional time-frequency maps. Compared to single CNN branch algorithms, MF-CNN shows improved decoding accuracy and has potential applications in motor function rehabilitation training after stroke. Wang et al. proposes an approach that combines an improved lasso with relief-f for feature extraction and selection in EEG signals. The method effectively extracts wavelet packet entropy features and topological features of brain function network, leading to high classification accuracy. Experimental results on two public EEG datasets demonstrate the effectiveness of this approach, and the average classification accuracy aboves 90%. This technology has potential applications in MI-BCI medical, rehabilitation, and other fields. Chen et al. proposes a layered spindle detection algorithm that combines the Morlet wavelet, root mean square (RMS) method, and an improved k-means algorithm to improve the accuracy and speed of detecting spindles during sleep. The algorithm demonstrates better performance stability, achieving higher precision, recall, specificity, accuracy, and F1-score compared to other methods. It provides an effective tool for automatic spindle detection and improves detection efficiency. Li et al. proposes a coherence-based graph convolutional network (C-GCN) method for extracting features and functional connectivity information from EEG signals in a MI-BCI. The C-GCN method achieves reliable and stable classification performance, with a maximum accuracy of 96.85%. The analysis of EEG data from SCI patients and healthy subjects provides an effective theoretical basis for the rehabilitation treatment of SCI patients. Xu et al. proposes a method for analyzing EEG signals during sleep stages using phase-locked value (PLV) to construct a functional connection network and investigates brain interaction. The α frequency band (8–13Hz) achieves the best classification effect with an accuracy of 92.59%. The proposed algorithm enhances sleep staging performance and promotes the development of EEG sleep staging systems. Tang et al. presents a modified sequential backward floating search (SBFS) approach for channel selection in MI-BCIs. The proposed method improves the time complexity of SBFS by selecting symmetrical channel pairs based on the EEG channel map. Experimental results on four BCI datasets demonstrate that the SBFS method achieves higher classification accuracy compared to using all channels or conventional MI channels, outperforming state-of-the-art selection methods. Du et al. proposes a dual attentive fusion model (DAFM) for EEG-based BCI classification. Experimental results on four datasets demonstrate that the proposed method outperforms state-of-the-art approaches, highlighting the effectiveness of the DAFM in enhancing feature expression. Yang et al. explores effective EEG features for recognizing different valence emotions. First-order difference, second-order difference, high-frequency power, and high-frequency differential entropy features performe well in emotion recognition. These findings provide valuable guidance for EEG-based emotion recognition feature extraction and selection. Wen et al. proposes future directions for cross-task EEG signal analysis research, including increasing sample size, exploring feature extraction and classification simultaneously, subdividing tasks, and investigating cross-task regression models. Conducting research in these areas can advance cross-task EEG analysis to a higher level.

In addition, the Research Topic is innovative in the motion paradigm of BCIs. Liu et al. proposes movement intention encoding paradigm based on sequential finger movement, showing potential for expanding the instruction set of MI-BCIs. Offline and online experiments are conducted, demonstrating the feasibility of the proposed paradigm. Xiao et al. presents a novel v-BCI paradigm using weak and small stimuli to achieve nine instructions, and demonstrates higher information transfer rate and feasibility for widespread use. Huang et al. presents a wireless group-synchronized neural recording system for real-time multi-subject BCI analysis, achieves high signal correlation, low noise, and high information transfer rate. Bai et al. presents a hybrid BCI system combining P300 and steady-state visually evoked potential (SSVEP) for improved spelling accuracy and speed. The implemented BCI achieves 94.29% accuracy and 28.64 bit/min information transfer rate (ITR) in online tests. Offline calibration tests demonstrate an accuracy of 96.86%.

Moreover, ther e are studies to find physiological phenomena in patients EEG. Zhu et al. explores effects of repetitive transcranial magnetic stimulation (rTMS) on functional connectivity in chronic insomnia disorder (CID) patients. Findings indicate potential biomarkers for predicting clinical outcomes and suggest rTMS can improve symptoms and optimize treatment. Promising evidence for future clinical trials.

Finally, this Research Topic also includes a human-computer interaction discourse paper. Zhao et al. suggest combining cognitive psychology with AI to develop computers capable of recognizing emotions, understanding human feelings, achieving dialogue and empathy. Three examples highlight potential and importance of AI in understanding human mental states:face attraction, affective computing, and music emotion.

In summary, there are large varieties among the included studies in this Research Topic. This Research Topic emphasizes the importance of AI system combined with cognitive psychology to the development of AI, and introduces potential and application value of AI in understanding and identifying human psychological state. These research results provide useful reference and guidance for the development of BCI technology, sleep problem diagnosis and management, emotion recognition, motor function rehabilitation and other fields.

## Author contributions

FX: Writing—review & editing. DM: Build the article framework. T-PJ: Polish and revise article. PX: Collect the accepted articles. MX: Summarize the accepted articles. All authors contributed to the article and approved the submitted version.

